# For whom does it pay to be a moral capitalist? Sustainability of corporate financial performance of ESG investment

**DOI:** 10.1371/journal.pone.0285027

**Published:** 2023-05-01

**Authors:** Mariya Gubareva, Zaghum Umar, Tatiana Sokolova, Valentina Antonyuk

**Affiliations:** 1 Lisbon School of Economics and Management (ISEG), Research Centre in Economic and Organisational Sociology (SOCIUS) / Research in Social Sciences and Management (CSG), Universidade de Lisboa, Lisbon, Portugal; 2 College of Business, Zayed University, Abu Dhabi, United Arab Emirates; 3 South Ural State University, Chelyabinsk, Russian Federation; 4 HSE University, Moscow, Russian Federation; 5 Department of Economic Theory, Regional Economics, State and Municipal Administration, South Ural State University, Chelyabinsk, Russian Federation; Universitat Jaume I, SPAIN

## Abstract

This paper analyzes the risk-return characteristics of socially responsible investing by employing a time-varying capital gain and Sharpe ratio analysis for various investment horizons. We employ the MSCI ESG (environmental, social and governance) leaders indices in ten markets encompassing Australia, Canada, Europe, Japan, UK, USA, China, India, Russia, and South Africa. Our sample ranges from 2007–2020. We document that ESG investments have very desirable return and hedging attributes for investors in these markets, and especially so in the USA and emerging markets.

## 1. Introduction

The last few decades have witnessed an ever-increasing trend towards integration of global financial markets across various asset classes as well as across various regions. A direct consequence of this heightened integration is a relative reduction in the diversification opportunities. Therefore, we saw a corresponding increase in investors searching for alternative assets that offer higher return or lower risk ([1–7] inter alia). Traditionally, financial factors, such as risk-return profile of an asset, were the main drivers determining the desirability of an asset. However, during recent times non-financial factors have also gained a lot of importance [8–12]. One such factor that stands out is an increased focus on the sustainability of investment and financing decisions that has led to the creation of a new asset category termed as Socially Responsible Investments (SRI). The eligibility of an asset into the SRI category is based on various parameters, among which the Environmental, Social, and Governance (ESG) leadership criteria are among the top parameters [13]. Moreover [14], document that during the last few years, investors widely accepted to forego financial performance in order to invest in accordance with their social preferences in SRI mutual funds, while [15] find that ESG climate-conscious equity mutual funds are among the top receivers of the net flows.

However, the literature documents divergent results on the performance of SRI funds. For instance [16], report underperformance of ESG portfolios. On the contrary, [17], and [18] document the higher performance of SRI funds. Moreover [19], documents the mitigating effect of Corporate Social Responsibility (CSR) on crash risk in diverse geographies and identifies optimal levels of CSR to minimize idiosyncratic risk for each region. [20] documents the financial effectiveness of ESG integration with mainstream active investment styles. [21] investigate how risky are SRI stocks of Central and Eastern European companies and conclude that the investigated SRI companies are less risky relative to the broader market.

This paper aims to contribute to this growing literature and answer the question: does it pay to be socially responsible and if yes–where exactly? To address this challenge, we look at social responsibility through the lens of ESG investments, analyzing the performance of ESG Leaders in the developed (Australia, Canada, Europe, Japan, UK, USA) and emerging markets (China, India, Russia, South Africa). We employ the MSCI ESG Leaders’ indices comprising the companies that have the highest ESG performance in these markets. It is worth noting that the MSCI ESG Leaders’ databases are vastly used by diverse researchers investigating the ESG role and impacts and, hence, the selection of the MSCI ESG Leaders’ indices for our research is thoroughly supported by the literature; see e.g., [3, 4, 22, 23]. Being interested in the long-term sustainability agenda, and not in the intraday and daily trading of ESG positions, we study the performance of the ESG investments through the prism of time-varying capital gains (CGS) [24, 25] and Sharpe ratios [26] for diverse investment horizons. In what concerns the adopted methodology, the capital gains (CGS) metric is widely used in finance and economics in such domain such as personal saving behavior [27], tax optimization [28], interest rate sensitivity [24, 25] among many others. This is a prominent, computationally simple, and robust method, allowing to study investments in diverse financial instruments such as bonds, real estate, equities, etc., over varying time horizons, which provides a necessary flexibility and comparability of results. In what concerns the Sharpe ratio, it expresses the relationship between risk and return and therefore can be used to compare different assets, e.g., stocks from different geographies, subject or not to EGS screening. The Sharpe ratio was originally proposed in 1966 [29]. It informs investors whether the potential return expected from an investment justifies the risks involved ([30], and the references therein). The Sharpe ratio can be used to evaluate the total performance of an aggregate investment portfolio or the performance of an individual stock. Being straight-away to calculate, it also does not involve unnecessary complexity of computation. That is why, why using the two complementary techniques, namely, CGS and Sharpe ratio, we do not resort to any specific software being the Microsoft Excel sufficient to perform our calculations of the performance of the ESG leaders in different countries.

Considered from the prism of the theories of capitalism, the rational of our research is aligned with the conceptualization of stakeholder capitalism as shareholder capitalism shaped by the voluntary commitments to and regulatory requirements of ESG disclosure [31]. This rethinking of stakeholder capitalism as the maximization of shareholder value subject to ESG-driven constraints aimed at protecting other stakeholders’ interests has attracted a lot of attention after the recent publication by the U.S. Securities Exchange Commission of a proposal outlining rules to enhance and standardize climate-related disclosures for investors [32]. With the growing interest in ESG consciousness and disclosure, it is expectable that corporate decision-making based on maximizing shareholder wealth may be substituted by decision-making maximizing shareholder welfare [33, 34]. Henceforth, analyzing the performance of ESG Leaders across diverse geographies, comprising the developed and emerging markets, our research contributes to the development of stakeholders´ capitalism theory, providing an empirical base for further analysis, especially of ESG role in creating shareholder value and of how this value creation differs across major developing and developed economies.

Our study contributes and extends the new strand of literature on the performance of ESG related investments. Although currently capitalism focuses mostly on profit maximization, this paradigm is already changing. Overall, investing shifts away from shareholder supremacy towards greater involvement of stakeholders, i.e., to moral capitalism. This paradigm shift has become accelerated during the Covid-19 pandemic [3, 35]. The recent rise in financial instruments under the ESG label, especially in the aftermath of the Covid-19 crisis, needs to be accompanied by stricter ESG oversight [36].

Our results show that USA and the emerging markets offer the highest capital gains and risk-adjusted returns. Thus, emerging markets are a good avenue for ESG investment. Our findings have implications for portfolio managers and policy makers. The positive attitude towards ESG investing results in positive returns, allowing for the mitigation of common financial risk by tailor-made hedge strategies, either for hedging climate and social risks [37] or for reducing the financial uncertainty of investment results as ESG instruments present safe-haven and hedging-asset features [23]. The ESG investments offer an alternative investment solution to withstand adverse timing and market behaviors.

The paper continues as follows. Section 2 presents Literature Review. Section 3 describes the data and methodology. Section 4 presents the empirical results and discussion followed by the conclusion section.

## 2. Literature review

A rapid growth of literature on the performance of the ESG-compliant investments is driven by changes in investor preferences regarding environment, sustainability, and societal aspects. A vast body of research has recently addressed the return and volatility dimensions of the ESG-screened financial instruments with focus on a comparative analysis of the performance of ESG assets vis-à-vis conventional investments. In this section, we provide a synthetic discussion of the recent research publications, which are relevant for our study.

[1] answer the question whether sustainable investment can yield better financial returns. The authors perform a comparative study of ESG indices and non-ESG MSCI indices from both emerging and developed markets over the period 2013–2017. In particular, they use a vector error correction model to investigate the volatility spillover between the conventional and sustainable indices and conclude that the conventional and sustainable indices are integrated and that there is a flow of information between these two parts of the investment universe. [1] find that there is no significant difference in the performance between traditional assets and sustainable indices and that the latter represent a good substitute to the former.

Addressing the possibility to employ ESG investments for hedging climate change news [37], present a procedure to dynamically change environmental risk. The authors perform a textual analysis of newspapers. They analyze and construct climate news data series. Using a mimicking portfolio approach, the authors design climate change hedge portfolios. They use ESG scores to model the climate risk exposure of the analyzed firms. [37] demonstrate that their approach allows constructing industry-balanced parsimonious portfolios that provide resilience vis-à-vis hedging innovations in climate news, indicating a prominent avenue for financial approaches to hedging environmental risk.

In their turn, [2] model investing compliant with ESG criteria. The authors advocate lower expected returns of green assets in equilibrium due to investors´ moral preferences in holding them as well as due to the hedge properties of these assets allowing to mitigate environmental risk. However, green assets are found to outperform when positive innovations are experienced by the ESG factor, reflecting changes in investors’ preferences for holding green financial instruments. ESG investments grow the most when the differences in preferences of the economic agents are the largest. [2] conclude that sustainable investing results in a positive social impact by turning firms greener and by moving real investment toward green companies.

[38] answer the question whether sustainability-conscious investors need to abdicate a part of their financial return, i.e., whether there is an additional cost for sustainable investment. The authors acknowledge that sustainable investment practices provide an additional emotional-value return in terms of contribution towards sustainability. The research employs daily data on the conventional and sustainability-based equity indices for the years 2013–2017. The authors compare the conditional correlation and volatility of conventional and sustainable indices resorting to a dynamic conditional correlation GARCH model. [38] conclude that investors may switch to sustainable investments without additional concessions regarding the risk-return binomial in the major developed and emerging markets analyzed in this study.

Following this strand of research [9], revisit the conventional versus sustainable investment dilemma during the COVID-19 pandemic. The research employs daily data on the conventional and sustainability-based equity indices from January 2011 to June 2020. The authors resort to the time-frequency-based Granger-causality test and study the interdependence between the sustainable and conventional equity indices prior and within the COVID-19 pandemic time interval employing the wavelet coherence and wavelet coherence phase-difference approaches. Their outcomes reveal a unidirectional causality from sustainability-based on conventional indices in the short-run, while for the medium- and long-run the authors report bi-directional causality relationship. [9] find that the coherence is particularly strong at low frequencies, i.e., large investment horizons, implying the long-run coherence with sustainability-based indices in the lead within the COVID-19 period.

[39] explore the impact of ESG disclosures on firm value and financial performance in the airline industry, analyzing, in particular, the influence of ESG score and financial health of firms. Using data from 38 airlines worldwide for 2009–2019, the authors observe that contributions to governance initiatives positively contribute to a firm’s market-to-book ratio. They find that a firm’s participation in ESG activities results in higher levels of financial efficiency of corporations. Therefore, the authors claim that their outcomes may help company managers to allocate the resources needed for ESG activities by adopting more efficient and robust approaches towards sustainability issues.

[10] investigate the linkage between the ESG ratings and financial performance of exchange-traded funds during the onset of COVID-19. The authors employ multivariate regression models and analyze the differences and relationship between the financial returns of ETFs and their Eco-fund ratings during the pandemic-related financial market crash in the first half of 2020. Their results imply that higher levels of sustainability performance of ETFs do not protect investments from financial losses during a severe market downturn and, therefore, highlight the weaknesses of current ESG scores and rating methods to provide an adequate assessment during COVID-19.

[12] analyze the overperformance of green stocks and bonds relative to non-green instruments in 2013–2021. Authors argue that high returns of green assets are a consequence of unexpectedly strong increases in environmental concerns and not high expected returns. They find that German green bonds have outperformed their conventional analogs while US green stocks outperformed conventional US stocks because of strengthening environmental concerns. Despite that outperformance, the authors advocate that lower expected returns for green stocks than for conventional are consistent with theory. [12] estimate the expected returns following two approaches: *ex ante*, using implied costs of capital, and *ex post*, using realized returns purged of shocks from environmental concerns and earnings. They conclude that the recent underperformance of US stocks is explained by a theoretically motivated green factor.

Focusing on the interdependence between the ESG presence and financial results in Turkish market [40], investigate the relationship between ESG practices and corporate financial performance. The authors try to answer the question whether ESG efforts affect the bottom line of corporations. They study the influence of ESG disclosures on the firm-level financial performance of companies listed on the Borsa Istanbul Corporate Governance Index (XKURY) in 2007–2017. The authors employ the corporate governance principles of the Capital Markets Board and Global Reporting Initiative as environmental indicators. This study contributes to the existing literature by exploring the effects of twenty independent ESG factors on the financial performance of corporations. In particular, the outcomes show a negative effect of environmental disclosure on corporate financial performance, while stakeholders´ involvement in management contributes to operational efficiency in the social dimension of ESG.

[41] revisit green and conventional finance spillovers in the post-COVID times. The authors investigate the causality and spillover effects between NASDAQ clean energy indices and their conventional analogs. The employed daily data sample covers the period from August 2011 to June 2021. The approach used in this study is based on Granger Causality test and the spillover models. The authors show that the overall connectedness between green and conventional indices increases after COVID-19 fueled meltdown in March 2020. Moreover, they argue that after this initial meltdown, fund managers and investors subtly commence to pay more attention to sustainable indices. [41], therefore, conclude that portfolio managers can promote portfolios that provide a high return and are environmentally sustainable.

Already addressing the applied aspects of green financing [42], provide a comprehensive literature review of transitioning green finance from theory to practice for renewable energy development. The authors select papers from 1990 to 2021 employing the “climate finance” and “green finance” keywords and analyze an extensive sample comprising 222 studies retrieved from the Web of Science and Scopus databases. They address several theoretical underpinnings of the constructs and themes uncovered through the results and identify current research trends, hotspots, and prospective research opportunities. Finally [42], highlight key topics in the field of green finance and recommend a four-part conceptual framework—goals, procedure, place, and perspective—based on the results of their survey.

As could be inferred from our concise literature review as well as from the literature survey by [42], a vast body of scientific research has been being produced regarding the issues of ESG investment performance and its integration with non-ESG financial markets. Among the top topics addressed by scholars, we observe a comparative analysis of ESG versus non-ESG investment performance, their overall connectedness, and the spillovers between these two parts of the investment universe. However, to the best of our knowledge, there is no research comparing financial performance of ESG investments across different countries, including emerging and developed markets. Our paper helps to fill this gap.

## 3. Data and methodology

Focusing on the corporate financial performance of ESG investment, we employ the MSCI ESG Leaders total-return indices, for ten representative geographies, comprising developed (Australia, Canada, Europe, Japan, UK, and USA) and developing (China, India, Russia, and South-Africa) economies. The MSCI ESG Leaders indices are USD-denominated free-float-adjusted market-capitalization-weighted metrics, designed to represent the performance of companies that have high ESG ratings relative to their sector peers, targeting an upper 50% ESG-wise representation. These indices are engineered to support common approaches to ESG investing, providing institutional investors with robust performance benchmarks.

The time series of the employed indices cover the 13-year-long period from September 2007 to December 2020. All data are obtained from Thompson Reuters DataStream database. We calculate the capital gain (CGS) from ESG investment in a chosen market as the difference between the initial and final price of the respective MSCI ESG Leaders index. We employ 3-, 6-, and 12-months-long holding period and generate the time series of the CGS with daily frequency, following previous research employing CGS metrics, see [24, 43, 44].

These price series enable us to quantify the capital gain relative to a chosen investment over any chosen interval as a difference between the respective index prices for holding period t to H:

$${CG}_{ESG\_Market}(t,H)=P_{ESG\_Market\_Index}(t+H)-P_{ESG\_Market\_Index}(t)$$ (1)

where *CG*_*ESG_Market*_(*t*, *H*) denote the capital gains (CGS) of the ESG index from t to t+H, *P*_*ESG_Market_Index*_ is the value of the ESG index at a given time. Eq (1) allows for calculating the time-varying capital gains *CG*_*ESG_Market*_, by rolling over the investment. The time-varying *CG*_*ESG_Market*_ series can be used to obtain the descriptive statistics of the CGS for each chosen market.

In order to account for risk-adjusted returns, we extend our econometric framework given by Eq (1) to incorporate in the Sharpe ratio [26] estimations for the three investment horizons mentioned above. Sharpe ratios (SR) are calculated as follows:

$${SR}_{ESG\_Market}(t,H)={(Average((R}_{ESG\_Market\_index})(t,H))-RF)/Std.dev({(R}_{ESG\_Market\_index})(t,H))$$ (2)

Where *SR*_*ESG_Market*_(*t*, *H*) denote the Sharpe ratio of the ESG leader index, (*Average*((*R*_*ESG_Market_index*_)(*t*, *H*))) is the average of the daily returns of ESG index from *t* to *t+H*, RF is the risk free rate and *Std*.*dev* ((*R*_*ESG_Market_index*_)(*t*, *H*)) is the standard deviation of the daily return of ESG index from *t* to *t+H*.

## 4. Empirical results and discussion

We use Eq (1) to compute the time-varying CGS for investment horizons of 3, 6, and 12 -months. Fig A-1 in S1 Appendix shows the distribution of the time-varying CGS. We notice a lot of variation in the CGS, underscoring our premises of calculating the time-varying CGS. Furthermore, we notice that the capital gains exhibit a spike after the global financial crisis as well as during the Covid-19 pandemic. In order to gain further insight, we focus on the distributional characterises of the time-varying CGS and report in Table 1 the main statistics of for each index. We notice that USA, Russia, China, India, and South Africa offer sizable higher mean CGS for all investment horizons. The standard deviation of the CGS is higher for emerging markets reflecting higher risk, with the highest standard deviation for Russia. The higher order moments, such as skewness and kurtosis are high, underscoring asymmetry and fat tails for almost all indices. It’s interesting to note that the highest CGS are earned in emerging market economies except for USA. Thus, our results allow claiming that the bigger social inequality in a country, the better is the ESG investment performance. Perhaps a plausible reason for this is that people subject to high inequality conditions could better feel the real beneficial impact of such investments in comparison to advanced economies, where reasonable equality conditions are already in place. Hence, in less privileged countries, a growing necessity in ESG investments provides positive feedback to the so-called victorious (opposite to vicious) cycle. As supporting evidence, we invite our readers to consider the superior performance of the emerging markets versus developed ones.

**Table 1:** Descriptive statistics for capital gains from ESG investments, 2007–2020.

	Australia	Canada	China	EU	India	Japan	Russia	S_Africa	UK	USA
**3-month CGS**										
Mean	1.64	1.73	2.91	1.57	2.39	1.26	3.61	1.99	0.59	2.55
Median	2.47	2.61	3.50	2.51	2.92	2.05	3.71	2.59	1.60	3.74
Maximum	56.08	66.42	45.34	58.29	102.92	38.95	177.96	72.07	48.82	42.30
Minimum	-45.85	-53.84	-53.87	-45.85	-50.02	-35.64	-80.23	-47.70	-43.41	-42.01
Std. Dev.	12.83	11.76	12.37	11.26	13.81	8.56	23.19	13.84	9.98	8.44
Skewness	0.02	-0.41	-0.33	-0.28	0.84	-0.34	1.04	0.09	-0.49	-0.95
Kurtosis	5.34	7.53	3.74	5.63	10.98	4.3	12.22	4.99	6.17	6.97
**6-month CGS**										
Mean	1.47	1.61	2.77	1.49	2.20	1.09	3.74	1.80	0.52	2.46
Median	2.35	2.46	3.29	2.39	2.59	1.82	3.90	2.39	1.50	3.65
Maximum	56.08	66.42	45.34	58.29	102.92	38.95	177.96	72.07	48.82	42.30
Minimum	-45.85	-53.84	-53.87	-45.85	-50.02	-35.64	-80.23	-47.70	-43.41	-42.01
Std. Dev.	12.85	11.81	12.44	11.33	13.86	8.55	23.34	13.84	10.04	8.49
Skewness	0.03	-0.39	-0.3	-0.27	0.88	-0.32	1.03	0.11	-0.47	-0.93
Kurtosis	5.38	7.51	3.7	5.58	11.06	4.34	12.12	5.04	6.12	6.9
**12-month CGS**										
Mean	6.73	5.60	12.64	6.24	9.96	4.90	12.34	8.57	2.79	10.94
Median	4.46	6.50	11.33	4.87	8.97	6.77	3.52	6.42	1.04	13.67
Maximum	138.59	98.06	114.59	92.70	153.87	55.78	289.78	148.33	80.33	81.58
Minimum	-59.49	-54.13	-67.42	-58.10	-62.70	-43.92	-82.11	-59.33	-55.73	-47.20
Std. Dev.	26.07	21.39	25.69	21.36	29.52	15.36	50.30	27.39	19.02	16.85
Skewness	1.1	0.38	-0.04	-0.27	1.27	-0.55	1.83	0.76	-0.22	-0.9
Kurtosis	7.09	5.15	3.24	4.15	7.76	3.79	9.16	4.87	4.6	5.69

Note: All values are in (%) except for skewness and Kurtosis. All average CGS are statistically significant at 1%.

In addition, the better is the social side of life, paradoxically or not, the worse in the profitability of ESG investments. As supporting evidence, we highlight UK, Japan, Canada, and EU excluding UK (Note: According to Social progress index ranking, they are ranked Australia, Canada, Japan, major EU countries are ranked tier 1, USA and UK are ranked tier 2, Russia is ranked Tier 3, South Africa and China are ranked Tier 4 and India is ranked Tier 5. https://www.socialprogress.org/index/global/results). There could be cogitated two different interpretations related to an important dichotomy. On the one hand, the highest proportion of asset owners in these geographies is probably already active in ESG space, resulting in a current overinvestment in the ESG-conscious companies. But, on the other hand, the low performance of ESG investment could also indicate a high potential future growth for the ESG investments in these regions.

Next, we extend our analysis and analyse the risk-adjusted returns by computing the time-varying Sharpe ratios using Eq (2). The time-varying Sharpe ratios are shown in Fig A-2 in S1 Appendix. Similar to the CGS, we notice that the Sharpe ratios also exhibit a lot of variation over the sample period, therefore, underscoring our premise of computing time-varying Sharpe ratios. We focus on the distributional characterises of the time-varying Sharpe ratios and report in Table 2 the main statistics of for each index Sharpe ratios for 3, 6, and 12 months investment horizons. Here again, we notice that USA exhibits the highest average Sharpe ratios. Again, the emerging markets have large average Sharpe ratios, which are equivalent or higher than the other developed markets. This supports our earlier notion of desirability of ESG investments in emerging markets. The standard deviations of the distribution of Sharpe ratio are comparable for all indices. Similarly, as expected, we notice that the distribution of Sharpe ratios is more symmetric with lower skewness and kurtosis values.

**Table 2:** Sharpe ratio for 3, 6, and 12-month periods, 2007–2020.

	AUSTRALIA	CANADA	CHINA	EU	INDIA	JAPAN	RUSSIA	S_AFRICA	UK	USA
3-month SR										
Mean	0.03	0.04	0.05	0.04	0.04	0.03	0.04	0.03	0.02	0.07
Median	0.04	0.05	0.05	0.04	0.04	0.03	0.04	0.03	0.03	0.07
Maximum	0.45	0.38	0.39	0.34	0.41	0.36	0.39	0.33	0.33	0.42
Minimum	-0.29	-0.35	-0.29	-0.31	-0.29	-0.34	-0.34	-0.31	-0.32	-0.23
Std. Dev.	0.12	0.12	0.12	0.12	0.12	0.10	0.12	0.10	0.11	0.11
Skewness	0.06	-0.05	0.05	-0.16	0.02	0.04	-0.06	0.03	0.00	0.11
Kurtosis	2.60	2.72	2.36	2.54	2.54	2.85	2.67	2.80	2.61	2.80
6-month SR										
Mean	0.03	0.03	0.04	0.03	0.04	0.03	0.03	0.03	0.02	0.06
Median	0.03	0.03	0.04	0.03	0.03	0.02	0.03	0.03	0.02	0.06
Maximum	0.28	0.28	0.28	0.26	0.30	0.23	0.26	0.26	0.23	0.28
Minimum	-0.19	-0.18	-0.20	-0.22	-0.21	-0.16	-0.24	-0.18	-0.17	-0.14
Std. Dev.	0.08	0.08	0.08	0.08	0.09	0.07	0.08	0.07	0.07	0.07
Skewness	0.33	0.08	0.09	0.03	0.24	0.21	0.07	0.05	0.12	-0.05
Kurtosis	2.73	2.64	2.60	2.60	2.74	2.65	2.52	2.84	2.56	2.57
12-month SR										
Mean	0.02	0.03	0.04	0.03	0.03	0.02	0.03	0.02	0.02	0.05
Median	0.02	0.03	0.04	0.02	0.03	0.02	0.02	0.02	0.01	0.05
Maximum	0.19	0.18	0.22	0.19	0.21	0.15	0.16	0.18	0.14	0.21
Minimum	-0.10	-0.11	-0.12	-0.14	-0.12	-0.11	-0.13	-0.11	-0.12	-0.12
Std. Dev.	0.05	0.05	0.06	0.06	0.06	0.04	0.06	0.05	0.05	0.05
Skewness	0.15	0.02	0.22	0.13	0.07	-0.05	0.17	0.11	0.09	-0.08
Kurtosis	2.77	2.70	2.95	2.78	3.07	2.66	2.06	2.83	2.42	3.02

Note: All average Sharpe ratios are statistically significant at 1%.

An important finding of our research is that the Sharpe ratios for the ESG stocks in the United States are higher than for the rest of the considered countries, making the ESG investments in the US markets by far the most advisable from ESG conscious investors´ point of view. The possible explanation of this fact is that the US investors are rather ESG doers, even though currently there is a relatively hostile political climate for fully embracing the sustainability agenda. E.g., in accordance with [45], the ESG criteria in the USA take the third place after considering, firstly, financial metrics and, secondly, geopolitical factors. However, ESG investing in the USA seems fairly attractive for moderate ESG investors, as good returns are obtained within a rather moderate volatility. It is in line with [19] concluding that ESG investments in the USA have a mitigating effect on crash risk.

It is worth mentioning that demand effects play an important role in explaining the observed performance of ESG stocks [2]. Therefore, the observed overperformance of ESG firms might be driven by changes in investors’ preferences regarding sustainability as well as environmental and governance-related dimensions. This also implies that one cannot say much about the drivers of observed over- or underperformance by simply looking at the cross-section of realized returns. Although the time dynamics of investors´ preferences vis-à-vis ESG aspects of their investments across different geographies stays outside of the scope of the current research, we would like our reader, be aware of the importance of demand effect at play underlying the differences in performance of the ESG investments in diverse countries.

It is worth noting that there exist some attempts in the literature to address the role of the demand channel in the ESG performance with focus on the US market. For instance [46], study the influence of green sentiment on stock returns and corporate behavior. They propose a new method to estimate non-fundamental demand shocks for green financial assets based on the arbitrage activity of exchange-traded funds and find that over the period 2010–2020, shifts in green sentiment anticipate a persistent stock-price out-performance of more environmentally responsible firms. [12] dissect green return of German green bonds and US green stocks and show that both outperform the respective non-green, so-called brown counterparts. However, the authors claim that such performance reflects unexpectedly strong increases in environmental concerns, not high expected returns. The authors argue that despite outperformance, lower expected returns for green stocks than for brown are consistent with theory. Moreover [12], show that a theoretically motivated green factor explains much of value stocks’ recent underperformance. In its turn [47], through the joint practice of exclusionary screening and environmental, social, and governance (ESG) integration, demonstrates how sustainable investing affects asset returns. The author develops an asset pricing model with partial segmentation and heterogeneous preferences. Focusing on US stocks, he estimates the model by applying it to sin stocks as excluded assets and using the holdings of green funds to proxy for environmental integration. The average annual exclusion effect is 2.79% for the period 1999–2019. However, the three above mentioned studies present certain drawbacks and are focused only on the US market, without addressing the cross country differences. Therefore, further research along this demand-channel strand, investigating the influence of demand on the ESG investment performance across diverse countries and regions is highly desirable.

The above analyses document the performance of ESG investments on a standalone basis. To present a comparative analysis of the performance of ESG assets vis-à-vis conventional investments, we consider a portfolio of an investor who invests in only conventional equities net of the ESG equities computed as the difference between the return on the MSCI country equity index and MSCI ESG leaders index. This portfolio allows us to answer whether an investor is better off by excluding ESG investments from the portfolio (higher Sharpe ratios for this portfolio) or vice versa (lower Sharpe ratios). Table 3 reports the summary statistics for this portfolio. We notice from Table 3 that the average Sharpe ratios for the conventional investor are lower than those for an ESG investor discussed in Table 2. The time-varying Sharpe ratios reported in Fig A-3 in S1 Appendix, also supports this conclusion.

**Table 3:** Sharpe ratio for 3, 6, and 12-month periods, 2007–2020.

	AUSTRALIA	CANADA	CHINA	EU	INDIA	JAPAN	RUSSIA	S_AFRICA	UK	USA
3-month SR										
Mean	0.01	0.00	-0.02	0.00	-0.02	0.00	-0.02	-0.01	0.01	-0.03
Median	0.01	-0.01	-0.02	-0.01	-0.01	0.00	-0.02	0.00	0.00	-0.03
Maximum	0.28	0.26	0.30	0.43	0.22	0.29	0.32	0.34	0.32	0.19
Minimum	-0.25	-0.21	-0.27	-0.29	-0.34	-0.31	-0.38	-0.40	-0.25	-0.27
Std. Dev.	0.08	0.07	0.10	0.11	0.08	0.09	0.10	0.11	0.08	0.07
Skewness	0.21	0.29	0.19	0.57	-0.50	0.00	0.10	-0.10	0.11	-0.31
Kurtosis	3.20	3.20	2.95	3.77	3.66	2.88	3.15	2.65	2.93	3.22
6-month SR										
Mean	0.01	0.00	-0.02	-0.01	-0.01	0.00	-0.02	-0.01	0.01	-0.03
Median	0.01	0.00	-0.02	-0.02	-0.01	-0.01	-0.02	0.00	0.00	-0.03
Maximum	0.14	0.18	0.20	0.32	0.16	0.18	0.22	0.19	0.18	0.11
Minimum	-0.14	-0.13	-0.20	-0.23	-0.23	-0.17	-0.19	-0.26	-0.16	-0.19
Std. Dev.	0.05	0.04	0.07	0.08	0.06	0.06	0.07	0.07	0.06	0.05
Skewness	-0.22	0.68	-0.01	0.87	-0.64	0.12	0.30	-0.20	0.07	-0.33
Kurtosis	3.05	5.00	3.01	4.82	3.66	2.31	2.52	2.52	3.01	3.17
12-month SR										
Mean	0.01	0.00	-0.02	-0.01	-0.01	0.00	-0.01	-0.01	0.00	-0.03
Median	0.01	0.00	-0.02	-0.02	-0.01	-0.01	-0.01	-0.01	0.01	-0.03
Maximum	0.09	0.08	0.07	0.23	0.09	0.11	0.12	0.11	0.11	0.06
Minimum	-0.09	-0.07	-0.16	-0.12	-0.13	-0.09	-0.13	-0.17	-0.10	-0.12
Std. Dev.	0.03	0.02	0.04	0.05	0.04	0.04	0.05	0.05	0.04	0.03
Skewness	-0.21	0.47	-0.57	1.40	-0.42	0.42	0.04	-0.05	-0.22	-0.22
Kurtosis	2.95	3.88	3.00	6.43	2.72	2.68	2.14	2.42	2.42	2.69

Note: All average Sharpe ratios are statistically significant at 1%.

At this point, it is worth mentioning that our results corroborate several previous research outcomes. For instance, our comparative analysis of the performance of ESG assets vis-à-vis conventional investments are in line with the [1] results, evidencing that despite of some expectable differences in the performance of traditional assets and sustainability-screened financial instruments the latter represent a good substitute to the former. Both metrics used in our study, namely, capital gains and Sharpe ratios, vary with the duration of the investment horizon, along the time, and across the countries, thus, echoing the conclusion by [48], who evidence that the investment in green energy fuels a sustainable green economy, but acknowledges that its effectiveness varies from country to country. And finally, yet importantly, our results corroborate [38] conclusion that currently investors may switch to sustainable investments without additional concessions regarding the risk-return binomial in the major developed and emerging markets. It is possible due to a strong contemporaneous increase in environmental concerns of investors and consumers [7, 12] that has been driving higher returns of ESG assets vis-à-vis conventional financial instruments.

## 5. Conclusions

We study the investment performance of firms that are industry leaders in terms of their ESG ranking, employing Sharpe ratios and CGS analysis. For the period 2007–2020, we employ the MSCI total-return indices for ESG leaders in 10 different developed and developing countries and analyze risk-return characteristics for different investment horizons.

Our findings are five-fold. First, we find that the investigated ESG Leaders´ companies from the selected geographies exhibit different risk return attributes and ESG asset dynamics clearly evidences asymmetries in risk and return patterns. Second, we document that the Sharpe ratio for the ESG stocks in the United States is higher than for the rest of the considered countries, making the ESG investments in the US markets by far the most advisable from ESG conscious investors´ point of view. Third, emerging markets exhibit better capital gains compared to most of the developed markets except USA. Russian market attractive for aggressive ESG investors, as it presents higher average returns, however, also with the highest standard deviation, meaning investors should mind the risk. Fourth, the bigger social inequality in a country, the better is the performance of ESG investments. Fifth, the more advanced is socio-economic side of life, the weaker are the returns of ESG investments, as inferred from the low returns observed for investments in EU, Japan, Canada, and UK.

In respect to our contribution to the existing theories of capitalism, we highlight that our research corroborates with the vision of stakeholder capitalism as the ESG-constrained shareholder capitalism. ESG disclosures make corporate agents recall the necessity of a more meaningful approach to how their corporations act along the three ESG dimensions. Therefore, the proposed by the U.S. Securities Exchange Commission enhancement of climate-related disclosures supports a profound move from shareholder to stakeholder capitalism. Answering the question for whom it pays to be a moral capitalist, we address the cross-market differences in this fundamental passage between the two capitalisms and help to reach a better understanding of ESG role in shareholder value creation across different geographies.

Our results confirm that ESG investments are desirable investment options in terms of both returns and hedging attributes. Our research is potentially helpful for SRI practitioners and policy makers promoting a new form of environment-conscious and socially beneficial investment, becoming nowadays widely known as moral capitalism. Our findings have important practical and managerial implications. Our outcomes provide an answer whether an investor is better off by excluding ESG investments from the portfolio (higher Sharpe ratios for this portfolio) or vice versa (lower Sharpe ratios), allowing therefore better portfolio allocation strategy. As evidenced by us, the performance of ESG leaders per geography is not stable and varies with time. Therefore, it is advisable for investors and portfolio managers to continuously monitor the relative performance of the ESG stock in order to be able to effectuate timely changes in relative weights of their investments. And finally, yet importantly, we provide an analysis of which ESG markets are overperforming, thus, providing a clue for investors, market practitioners, and investment managers regarding geographies offering superior performance of ESG conscious investments.

In what concerns the limitations of our study, we mention a restricted geographic coverage of our research and a rather limited historical depth of the employed time series. These opportunities for a more complete investigation will be addressed in future research. As it is mentioned during the discussion of the results, the demand effects play an important role in explaining the observed performance of ESG stocks. Therefore, a dedicated analysis of ever-changing investors´ preferences regarding sustainability and environment represents a prominent research domain for advanced studies on the subject matter. Hence, further research of differences in ESG performance and their drivers is highly desirable and will be duly addressed in future studies.

## Supporting information

S1 Appendix Supplementary figures. (DOCX)

S1 Data (XLSX)
