# Supplementary material for: For whom does it pay to be a moral capitalist? Sustainability of corporate financial performance of ESG investment
Source: PLoS One. 2023 May 1;18(5):e0285027. doi: 10.1371/journal.pone.0285027 (PMC10150969; doi:10.1371/journal.pone.0285027)
Supplement: S1 Appendix — (DOCX) [file pone.0285027.s001.docx]

**Appendices:**

**Figure A-1: Time-varying capital gains (CGS)**

**3_month CGS**

**6-Month CGS**

**12-Month CGS**

Note: This figure shows the time-varying capital gains for various ESG indices for 3-month, 6-month and 12-month investment horizons.

**Figure A-2: Time-Varying Sharpe Ratios for ESG investor**

**3_month Sharpe ratio**

**6-Month Sharpe ratio**

**12-Month Sharpe ratio**

Note: This figure shows the time-varying Sharpe ratios for various ESG indices for 3-month, 6-month and 12-month investment horizons.

**Figure A-3: Time-Varying Sharpe Ratios for conventional investor**

**3_month Sharpe ratio**

**6-Month Sharpe ratio**

**12-Month Sharpe ratio**

Note: This figure shows the time-varying Sharpe ratios for various ESG indices for 3-month, 6-month and 12-month investment horizons.
